# Psychometric properties of martial art kendo players: a multicultural exploratory online questionnaire survey

**DOI:** 10.3389/fpsyg.2025.1595577

**Published:** 2025-09-09

**Authors:** Michael Spantios, Kei Kobayashi, Toshiya Murai, Hironobu Fujiwara

**Affiliations:** Department of Psychiatry, Graduate school of Medicine, Kyoto University, Kyoto, Japan

**Keywords:** mental health, cultural differences, resilience, mindfulness, sport

## Abstract

**Introduction:**

Mind-body unity, may form through stringent exercise and focused breathing. Brief participation may increase psychometric properties combatting mental health issues. We investigated how psychometric properties manifest within kendo practitioners (KP) internationally. Do they have increased psychometric properties compared to non-practitioners (NKP) due to kendo experience; our hypothesis was that they do.

**Methods:**

The psychiatric questionnaires were disseminated through email and social media with informed consent obtained. All self-report questionnaires had good internal reliability and consistency. The study was performed using a cross-sectional design and conducted so that participants could only move on to subsequent questions if they had provided answers for prior questions. 230 European participants, 349 Japanese participants were present throughout this study. Their group was the independent variable as participants were divided by whether they practiced kendo or not. We used multiple psychiatric questionnaires like K6 (depression), COPE (stress), CD-RISC (resilience), Rosenberg’s self-esteem scale, SOC (confidence in dealing with life) and Emotional Regulation to measure various psychometric properties. Inclusion criteria were that patients be 18 or older and do exercise at least once a week.

**Results:**

For statistical methods a Mann–Whitney U test, descriptive statistics, reliability analysis and post-hoc power analysis were conducted using SPSS and GPower. Significant differences between KPs and NKPs for SOC (*p*-value = 0.013*), CDRISC (*p*-value = <0.001**), K6 (*p*-value = 0.008**) and self-esteem (*p*-value = <0.001**) were noted. SOC, CDRISC and K6 were deemed significant here but not when separate analyses were conducted for European or Japanese participants. European data showed no significant difference and for Japanese data only self-esteem was significant (*p*-value = 0.03*).

**Discussion:**

Our results support that kendo can lead to increased psychometric properties compared to non-kendo. Result differences when split in to Japanese vs European could be attributed to cultural differences. These cultural differences could affect baseline levels of reported self-esteem within a population and could be why there was a discernible difference between Japanese and European participants. Sports lacking this mindfulness component do not provide the same prescribed benefits. However, the degree of impact pertaining to these results remains to be seen and should be further investigated before being applied in a clinical setting in the future.

## Introduction

Exercise is generally believed to be good for your health whether it is intense cardiovascular workouts or weightlifting. The benefits that doing sport as a form of exercise provides can be beneficial to the respiratory, metabolic and cardiovascular systems but multiple studies have also shown that mental wellbeing and cognitive function can improve in tandem (Callaghan, 2004). More specifically, improved attention, motivation and memory function (Fujiwara et al., 2019); (Mandolesi et al., 2018). FDG-PET (fluorodeoxyglucose Positron Emission Tomography) studies have supported these findings indicating that exercise can determine changes in metabolic networks that are related to cognition through measuring glucose levels and synaptic function (Huang et al., 2016).

What sets martial arts apart from other sports is its focus on the cultivation of the mind. Unlike western sports that emphasise competing and winning, martial arts emphasise self- control, self-improvement and knowing thyself (Binder, 2007). This stems from the teachings of Zen Buddhism where a calm and still mind often referred to as ‘zen’ is emphasised. In East Asian cultures this form of Buddhism believes that wisdom alongside compassion is essential and should be present in all aspects of everyday life (Nagatomo, 2020).

This zen ideal can be seen in the training methods of martial arts like kendo. Kendo is a type of martial art that practices with bamboo swords and shares similarities with fencing. A central tenet of kendo specifically is its ability to integrate both body and mind through intense training methods that are said to be examples of “Zen in action” (Oosterling, 2011). The zen like state that arises from the unity of the mind and body is composed of two parts, firstly the physiological benefits that exercise can provide such as the regulation of the respiratory and cardiovascular systems mentioned above and secondly, a type of focused breathing that is said to calm and refocus the mind. This type of breathing referred to as ‘diaphragmatic breathing’ has been shown to significantly decrease anxiety and stress via decreased cortisol levels and significantly increase attention span upon control group comparison (Ma et al., 2017); (Lutz et al., 2009); (Matousek et al., 2010). Mindfulness is a state that can be achieved through repeated production of this zen ideal and is referred to as the ability to observe sensations, feelings and thoughts as they happen while remaining impartial to them (Weder, 2022). A mindfulness strategy called mindful attention has been shown to produce less neural activity in the subgenual ACC, ventral ACC, ventral mPFC and medial orbito-frontal cortex during exposure to stressful scenarios (Taren et al., 2015). The production of mindfulness through kendo could therefore be important in terms of stress coping and the ability to respond to negative events calmly.

A common problem that many people face is the abundance of stressors in daily life, but routinely taking part in martial arts like kendo is said to improve stress coping ability (Chiodo et al., 2011) and improve self-esteem in participants (Daniels and Thornton, 1992). This idea was further reinforced in a recent study that stated how positive emotions and stress relief mediate the relationship between physical activity and well-being (Oh et al., 2025). Therefore, the current theory is that active participation even over short periods of time may help to increase psychometric properties even in people who have never previously participated thereby lowering perceived mental health issues and leading to improved clinical outcomes over time even for individuals on the spectrum (Moore et al., 2020).

In this study, we investigated the psychometric properties and associated mental health within kendo players that manifests through increased mindfulness and psychological resilience on an international scale. Other psychological components of kendo players and the effects of training on their mentality was also noted, including duration and frequency of training. This was achieved through an international questionnaire survey that was scored in relation to multiple psychometric tests. Exercise being beneficial to mental health has become a well-established theory in the psychiatric community (Callaghan, 2004; Chiodo et al., 2011; Moore et al., 2020). Our reason for conducting this study was to further explore the idea that kendo is beneficial to mental health and to establish the idea that sports that include a mental component are more effective at conferring specific benefits than other sports. Therefore, the aim of the present study is to investigate the psychometric properties and associated mental health in kendo players internationally and our initial hypothesis was that kendo practitioners have increased psychometric properties compared to non-practitioners, due to prior kendo experience.

## Materials and methods

### Participants

The European data had 166 men and 64 women making 230 in total. 197 of them who do kendo (147 men and 50 women) and 33 who do not. (19 men and 14 women). Average age of men was 40 years old with a minimum of 18 years old and maximum of 74 years old. Average age of women was 33.92 also with a minimum age of 18 but maximum age of 67 (see Supplementary Table 1).

The Japanese data had 179 men and 170 women making 349 participants in total. Of those 179 men, there was 102 who practiced kendo and 77 who did not. Of the 170 women, 55 practiced kendo and 115 did not. There was a total of 157 KPs and 192 non-KPs. Average age of the men was 34.96 years with a minimum of 18 years and maximum of 67. Average age of women was 26.19 years with a minimum of 8 years and a maximum of 73, so there was quite a broad range of ages present in this study overall (see Supplementary Table 1).

### Procedure and outcome measures

Multiple questionnaires each of which constitute a different psychometric test and measure a different aspect of behaviour were used in this study. All of them were self-report questionnaires. The study was performed using a cross-sectional design and carried out so that all questionnaires could be completed from an accessible website. For each questionnaire, the participants could only move on to subsequent questions if they had provided answers for prior questions. No incentives were provided either but participants were free to review and change their answers if they so desired. Both the Japanese versions and English versions of these questionnaires were used. The inclusion criteria were that you did frequent exercise (at least once a week) and that all participants be at least 18 years of age.

The questionnaires were sent out in November 2017, the data collated henceforth and then analysed in August 2022. This was an open voluntary survey so a convenience sample was used and the questionnaires’ contents were disseminated through email and social media such as Facebook advertisements. Cronbach’s alpha was the same for both English and Japanese versions of the questionnaires. The answers to the questionnaires and any personal information were stored in an Excel file that was only accessible to the author KK of this study, using a password lock.

The K6 scale (Kessler et al., 2003; Furukawa et al., 2008) which measures psychological distress stemming from anxiety and depression related symptoms. K6 is a shorter version of the K10 scale where there are only 6 questions, and each question is rated on a five-point Likert scale. There is a total of 24 points and the higher the score the higher the psychological distress. Cronbach’s alpha was calculated to be 0.85 meaning good internal consistency and reliability. Completion rate was 82.5% (288/349) for the Japanese data and 75.7% (174/230) for the European data.

COPE (Carver et al., 1989; Otsuka et al., 2008) which contains 28 questions and is used to measure effective and ineffective ways of coping with stress. This questionnaire is commonly used in healthcare to see how patients are coping with serious circumstances. There are 3 dominant coping styles: problem focused coping, emotion focused coping, and avoidant coping. The scores for the three coping strategies are often presented as averages and show which coping style an individual uses most frequently. Cronbach’s alpha was 0.74–0.82 and completion rate was 83.4% (291/349) for the Japanese data and 74.3% (171/230) for the European data.

The CD-RISC questionnaire (Connor and Davidson, 2003; Awano et al., 2020) which contains 25 questions and is designed to evaluate a person’s resilience in the face of adverse life events. It measures several different components of resilience: change, stress, discouragement, focus, unpleasant feelings and coping. A higher score often reflects a higher level of resilience with scoring ranging from 0 to 100. Cronbach’s alpha was high at 0.93 and completion rate was 89.4% (312/349) for the Japanese data and 83.0% (191/230) for the European data.

Rosenberg self-esteem scale (Rosenberg, 1965; Mimura and Griffiths, 2007) is a 10-question scale that measures self-worth by measuring an individual’s positive and negative feelings about themselves. It is measured using a 4-point scale from strongly disagree to strongly agree. The higher the score, the higher a person’s self-esteem is said to be. Cronbach’s alpha was 0.79–0.88 and completion rate was 82.2% (288/349) for the Japanese data and 75.7% (174/230) for the European data.

Sense of Coherence (Antonovsky, 1993; Togari and Yamazaki, 2005) is a questionnaire concerning how individuals view life and the resources they use to improve or maintain their health. It is made up of 29 lifestyle questions. 11 concern compatibility, 10 items measure manageability, and 8 items measure meaningfulness. The scale ranges from 1 to 7 for each question where 1 is “I never have this feeling” and 7 is “I always have this feeling.” Total scores can range from 29 to 203. Cronbach alpha was 0.84–0.89 and completion rate was 94.0% (328/349) for the Japanese data and 87.4% (201/230) for the European data.

Emotional Regulation Questionnaire (Gross and John, 2003; Yoshizu et al., 2013) has 10 items and measures how emotions are regulated. There are two methods: cognitive reappraisal which means to rethink a situation and expressive suppression which means to suppress your emotions. The answers are measured on a 7-point scale from where 1 is strongly disagree and 7 is strongly agree. Cronbach’s alpha was lower at 0.45 and completion rate was 86% (300/349) for the Japanese data and 78.3% (180/230) for the European data. Despite the low alpha, this questionnaire was still included due to the fact that emotional regulation is said to be one of the key benefits that martial arts can provide over time (Oh et al., 2025).

### Statistical methods used

SPSS version 21 and GPower version 3.1 were the only statistical programmes used (Faul et al., 2007). The statistical methods used were descriptive statistics which allowed us to see the spread of the data and non-parametric tests like the Mann-Whitney U test were also used. Specifically, an independent-samples Mann Whitney U test was chosen because it allows for the comparison between two groups with unequal sample sizes as was the case here. The total number of responses for each questionnaire were counted and the data of the participants who had any missing questionnaire data were removed in order to ensure completeness and produce the final data set that was then used for the analysis. This was done for both Japanese and European data sets. Prior to this, questionnaire completion rate for both Japanese and European data sets were calculated. A post-hoc power analysis was conducted using Gpower to see if the power of our results were sufficient for our current sample size. A reliability analysis was performed to calculate Cronbach’s alpha which indicates the reliability and validity of the questionnaires and a 95% confidence interval was used as standard. The alternative hypothesis was that there should be a difference in distribution of the data between these two groups for each of the questionnaires. The initial assumptions were that the mental and physical benefits associated with kendo are not culturally specific and should indiscriminately apply to everyone who practices.

### Ethical considerations

Informed consent was obtained from the subjects before completing the questionnaires. The study was approved by the Ethics Committee of the Kyoto University Graduate School and Faculty of Medicine and was conducted in accordance with the Declaration of Helsinki. Approval number: R1185-3.

## Results

### Main results of all data

**Figure 1:**
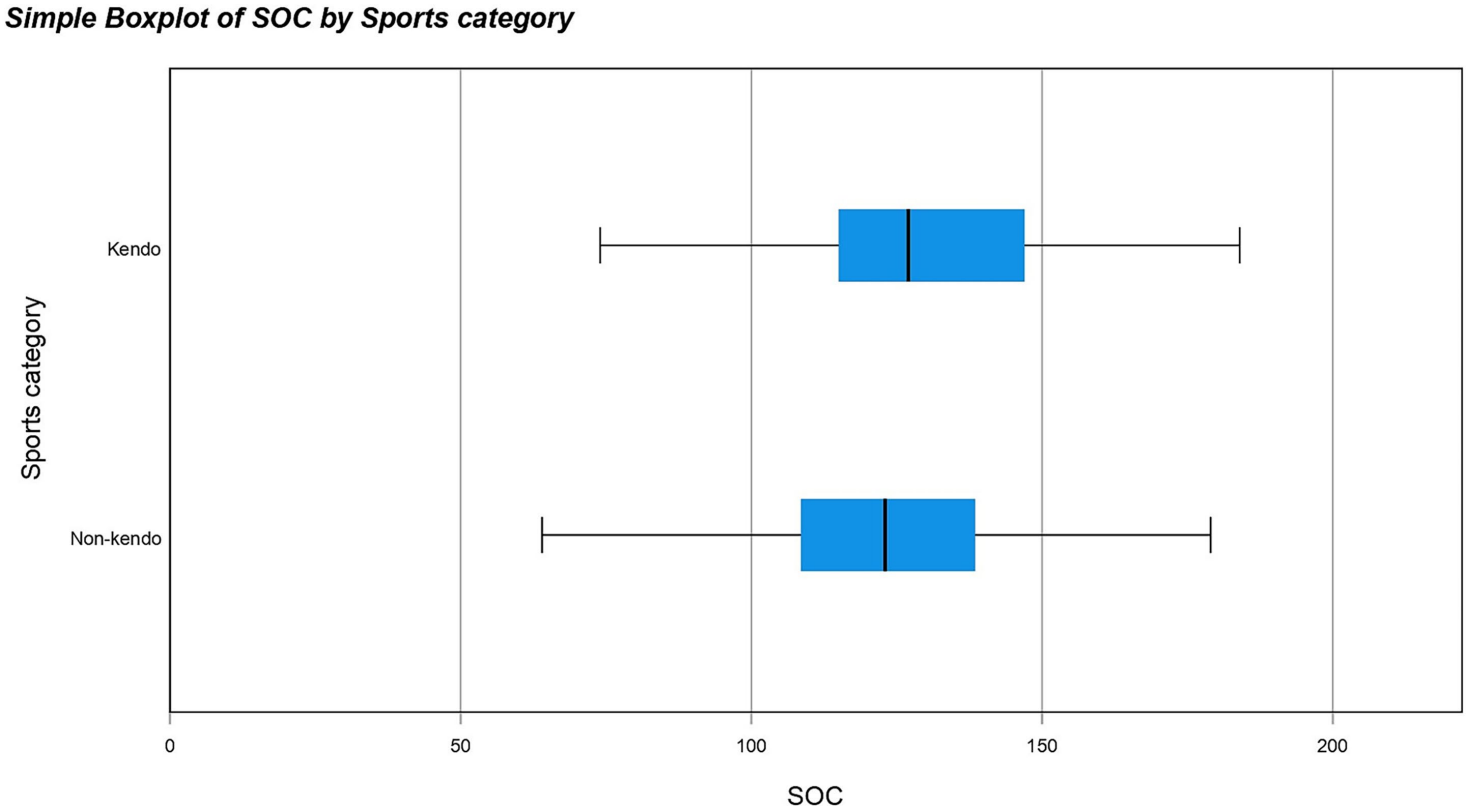
Box plot for SOC by sports category including outliers.

**Figure 2:**
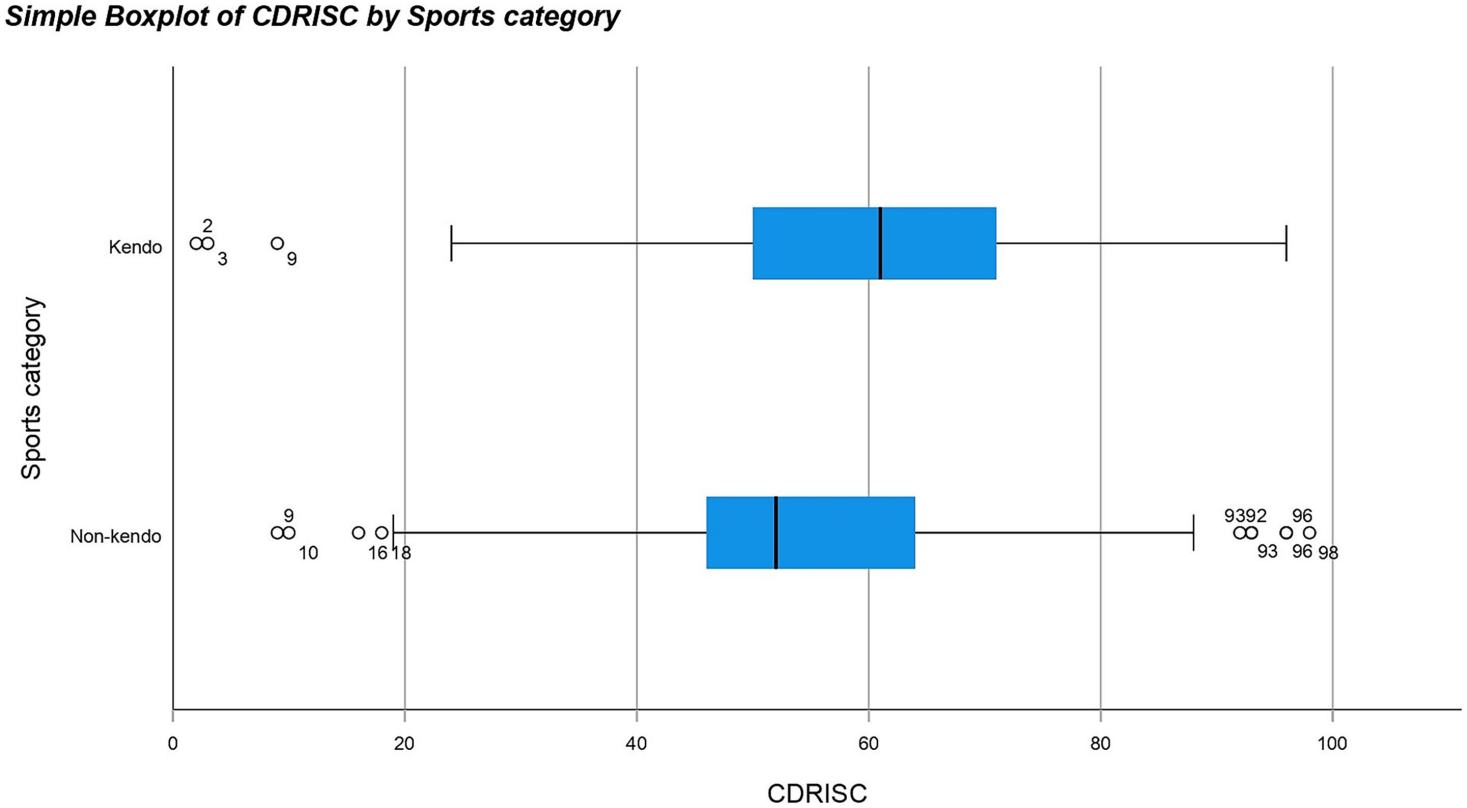
Box plot for CD-RISC by sports category including outliers.

**Figure 3:**
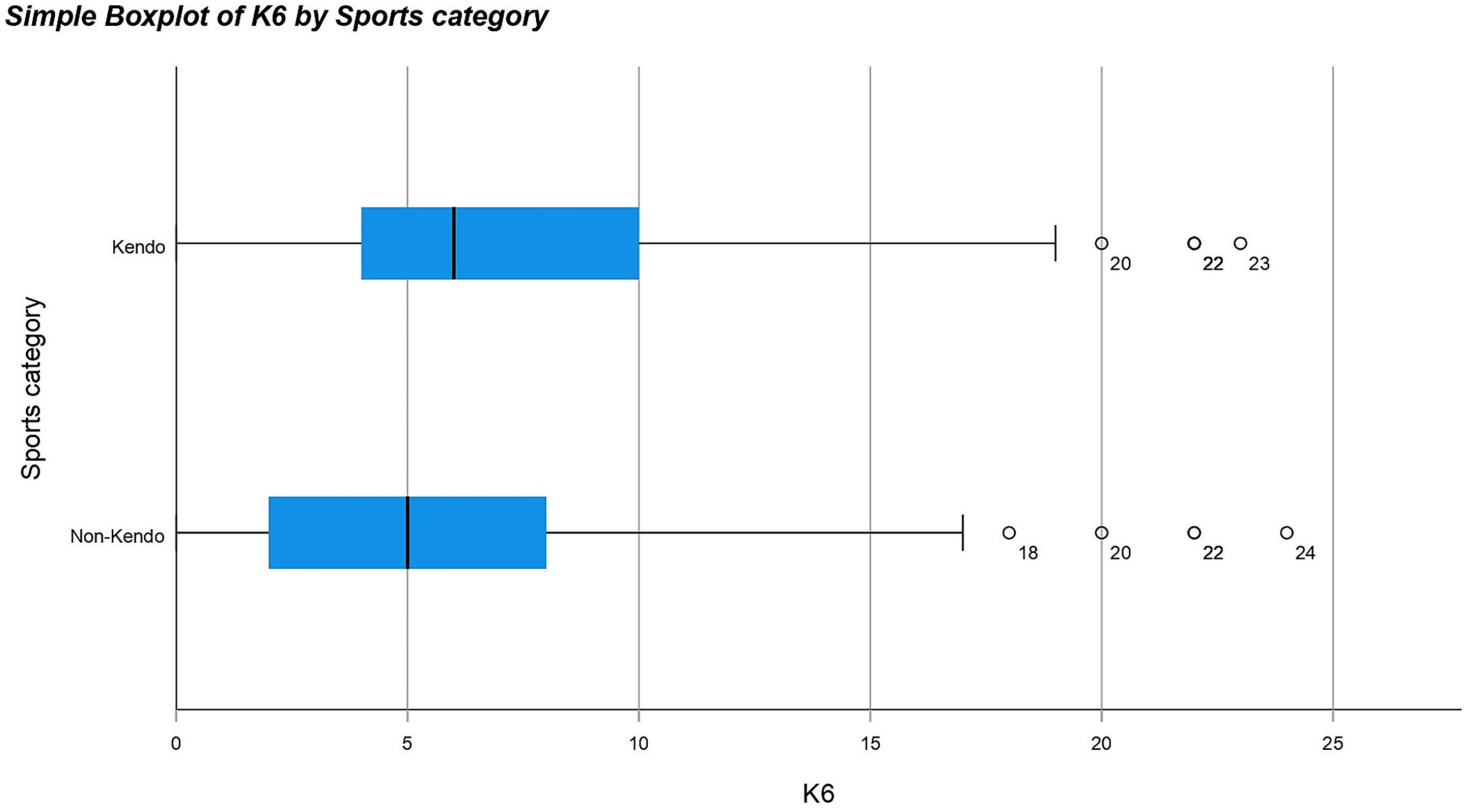
Box plot for K6 by sports category including outliers.

**Figure 4:**
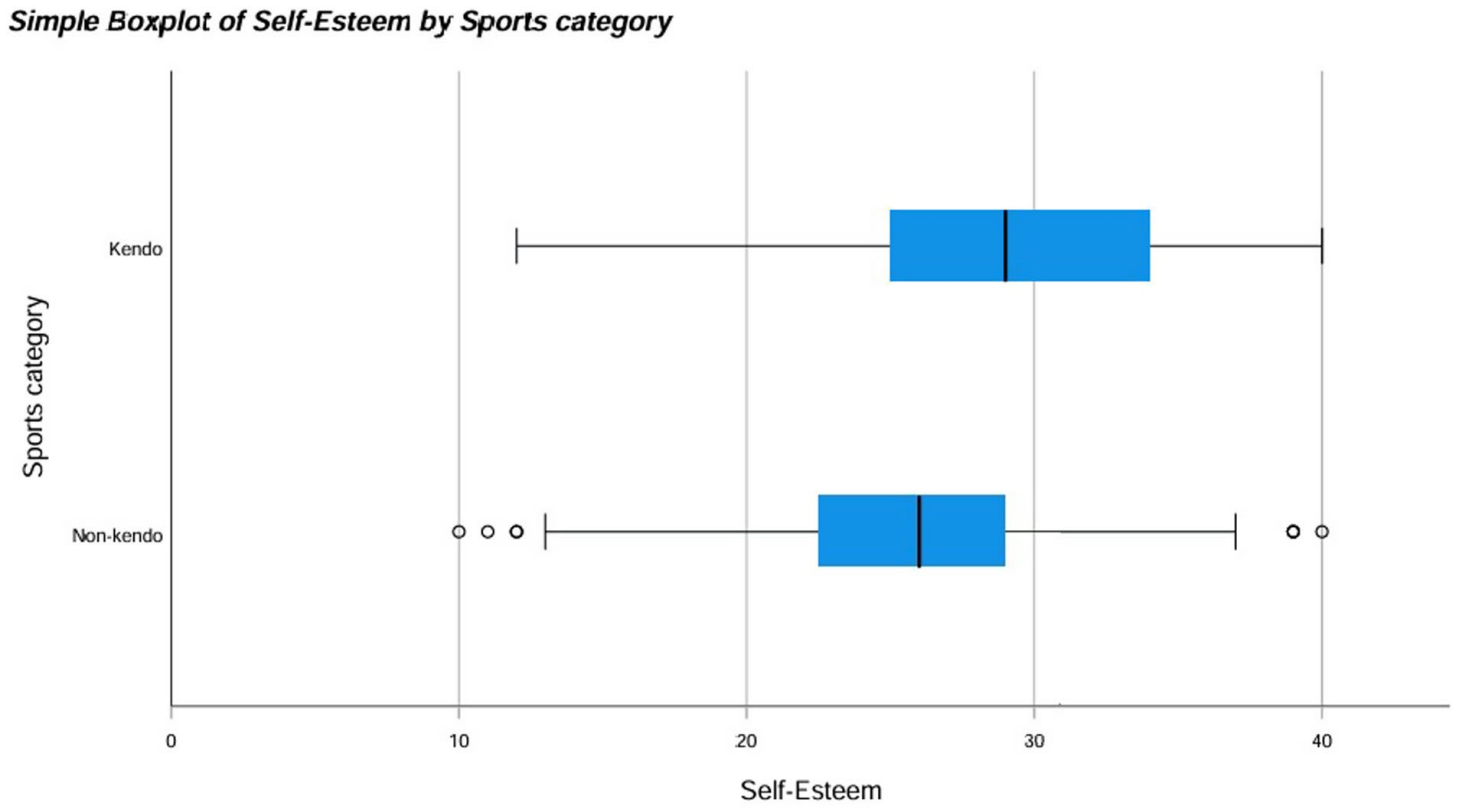
Box plot for self-esteem by sports category including outliers.

**Figure 5:**
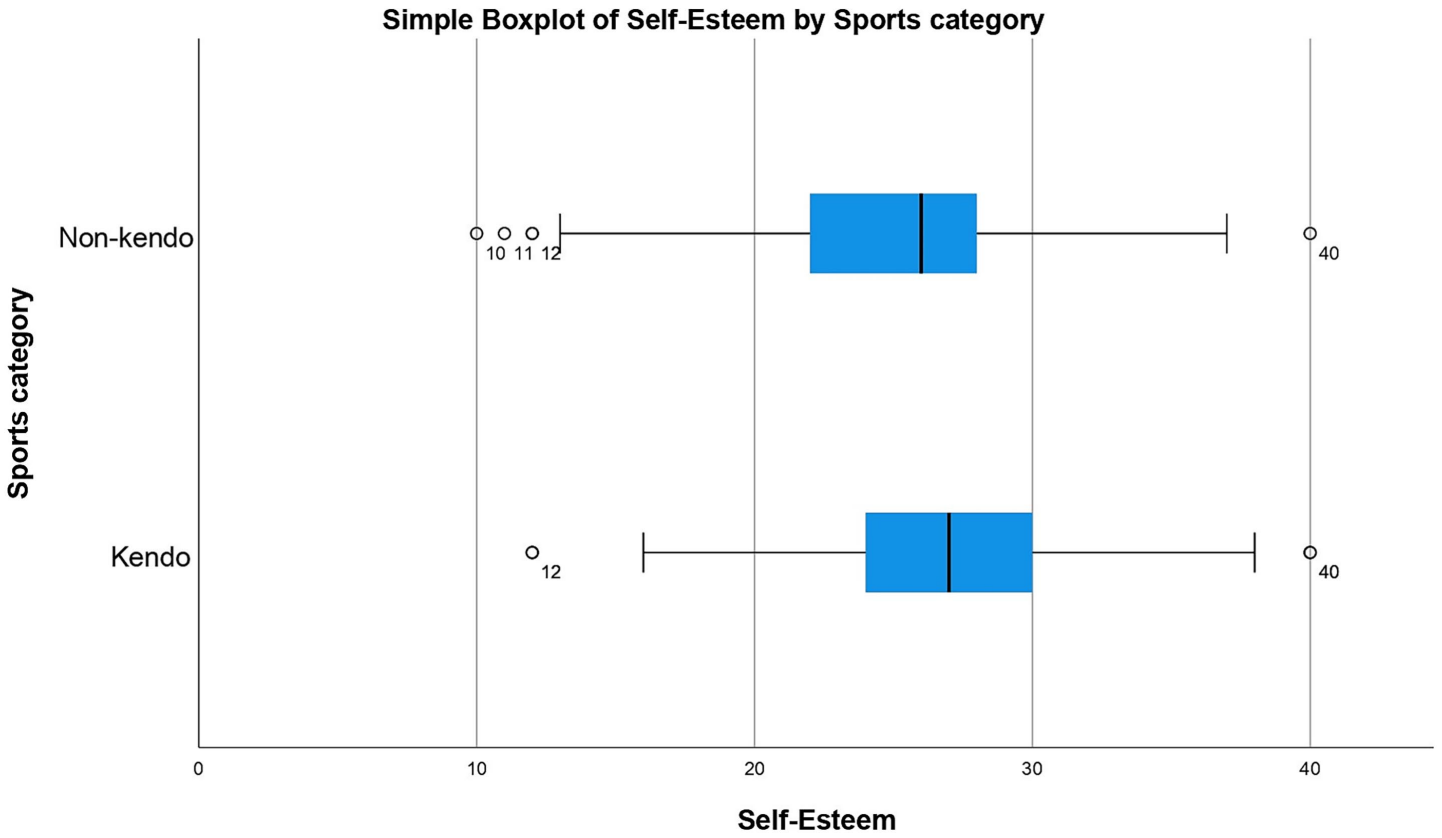
Box plot for self-esteem by sports category including outliers.

A non-parametric test was used because the results for both KP and NKP groups against all the different questionnaires were not normally distributed meaning a normal one-way ANOVA could not be used. The Mann Whitney U test revealed significant differences between the data distribution of SOC (*p*-value = 0.013*), CDRISC (*p*-value = <0.001**), K6 (*p*-value = 0.008**) and self-esteem (*p*-value = <0.001**) for KPs and NKPs with the medians for SOC, CDRISC, K6 and self-esteem being higher in KPs than NKPs (see Figures 1–4). The significance of all these questionnaires was surprising since they were not considered significant when either of the initial analyses were conducted for European or Japanese participants separately besides self-esteem (see Figure 5).

### European data results

A Mann Whitney U test revealed, there were no significant differences between the distribution of the data when it came to comparing these two groups for SOC, CD-RISC, Emotional Regulation, K6, Self-Esteem or COPE.

### Japanese data results

When the same analysis was conducted for Japanese participants, there were no significant differences between the distribution of the data when comparing KPs with NKPs for Sense of Coherence (SOC), CD-RISC, Emotional Regulation, K6, COPE. The only questionnaire that showed a significant difference between the median of the two groups was self-esteem with a *p*-value of 0.003 showing great significance (see Figure 5).

### Post-hoc power analysis results

The post-hoc power analysis we conducted using GPower was normal, two tailed, had a Cohen’s d of 0.5 and an alpha error of 0.05 as standard. The analysis showed that our sample size was plenty sufficient with an achieved power of 0.999 and a critical t of 1.964.

## Discussion

The purpose of this study was to determine whether kendo players have increased psychometric properties compared to non-kendo players and whether this could be applied on a global scale using multiple psychological questionnaires; potentially offering an alternative to passive therapy practices. Our hypothesis stated that kendo players have increased psychometric properties compared to non-kendo players and one of the underlying reasons could be due to intensive physical and mental training on a consistent basis (All Japan Kendo Federation, 2007). When all participants were included KPs on average had significantly higher scores in the CD-RISC, SOC, K6 and Rosenberg self-esteem questionnaires but no difference was found for the emotion regulation and COPE questionnaires. As can be seen from these results, KPs on average showed more resilience, confidence and had higher reported self-esteem than their NKP counterparts. Although depression scores were also higher in KPs, the overall low scores among the participants confirmed they were not clinically depressed individuals. When the Japanese participants were analysed separately only levels of self-esteem were considered to be significantly higher for KPs than NKPs.

As SOC assesses the way individuals view life and deal with stressors; a higher SOC score in this case would indicate more confidence in managing stressors and lead to a more positive outlook on life. This is a beneficial skill to develop as a common indicator in mental health disorders such as anxiety and depression is the feeling of overwhelm and a more negatively skewed outlook (Beevers et al., 2019; McLaughlin and Hatzenbuehler, 2009).

CD-RISC is the most commonly used scale for measuring psychological resilience. Being adept at dealing with adversity is a desirable skill as it allows one to approach situations calmly and more effectively manage them. As our data revealed that KPs were much more resilient than their NKP counterparts the intense physical training and mental clarity that characterises kendo may lead to increased mental resilience as a positive by-product.

For the Rosenberg self-esteem scale, our Japanese data indicated that there is a significant difference between the levels of self-esteem in individuals who practice kendo versus those who do not with the former having higher overall levels of self-esteem. The potential benefits of kendo, i.e., an increased sense of agency and accomplishment can activate the dopaminergic reward system during periods of intense training (Yu and Song, 2022). Continuous activation can lead to increased satisfaction about oneself or associated surroundings, thereby fostering a sense of heightened self-esteem over time. In turn, this can provide further incentive to keep training as the practitioners can see the fruits of their labour (Yu and Song, 2022).

As K6 is a mental health index most commonly used for indicating depression, the overall low scores of K6 among the participants confirmed that they were not clinically depressed individuals. A significant difference at the lower end of the scale without any specific or detailed interviews being conducted by psychiatrists, does not always indicate a clinically meaningful depression it simply states a level of distress rather than a specific diagnosis (Kim et al., 2016).

Upon considering all of these results, we noticed that our initial hypothesis has merit if we consider the data as a whole but differs when split into distinct groups (i.e., European vs. Japanese). We speculate that these results may be due to distinct cultural differences present between Japan and the associated European countries such as, but not limited to, differing levels of politeness, a reserved vs. relaxed nature and degree of independence (Heine, 2007). These differences can arise from being raised with either the collectivistic mindset of east Asian countries or the individualistic mindset of western countries and has the potential to affect the baseline levels of self-esteem within a population or at least how it is reported (Cai et al., 2007; Kurman, 2003). As we were looking to apply our results on a global scale looking at the differences between KPs and NKPs as a whole was more suitable.

As mentioned in the introduction the components of martial arts that are beneficial to your mental health are multiple. It allows for stress relief due to the promotion of mindfulness and practicing of diaphragmatic breathing as well as increased resilience due to continuously overcoming various mental and physical challenges (Zurita-Ortega et al., 2016). Another sport with martial art foundations that provides support both mentally and physically is judo. It can increase resilience like kendo as they too are constantly overcoming difficult challenges (Rossi et al., 2022). Judo can further provide the calmness of mind associated with martial arts by helping participants be less reactive to their surroundings and by giving them an increased sense of motivation (Rossi et al., 2022).

Sports without a martial arts basis such as football have not shown the same benefits. Recently, there has been an increase in the levels of anxiety and depression as well as burnout in professional footballers (Sarmento et al., 2021). The depressive symptoms ranged from 16.7 to 39% of surveyed footballers, both current and retired (Sarmento et al., 2021). However, during an eight-week randomised clinical trial, a group of retired footballers underwent mindfulness-based stress reduction therapy (MBSR) and it proved to be significantly beneficial as there was marked improvement in stress, depression and anxiety symptoms from baseline when compared to a control group who remained consistent throughout the trial (Norouzi et al., 2020). This is not to say that kendo players do not feel depressed or anxious during long bouts of intense training or stressful situations, simply that they may be more well equipped to process these emotions. Therefore, aspects of MBSR that are shared with martial arts like the ability to maintain concentration and increased mental clarity could potentially apply to other sports such as football and could be the next step in mental health awareness and facilitation in sport.

Mental health practitioners and policy makers can present these findings and any other corroborating evidence to hospitals and encourage them to set up mindfulness related rehabilitation programmes at daily clinical practices for individuals that have psychiatric illnesses, this can include physical activities like kendo. There is already a precedent for this through activities such as yoga which was initially seen as a fad but has now begun to be recognised as a valuable psychiatric treatment (Khalsa et al., 2013; Varambally et al., 2020; Koncz et al., 2013; Wu et al., 2025). On top of this, these practices can also be used as a tool for mental health promotion to the general public.

An initial limitation is age, as it is considered to be a confounding variable especially amongst the KPs’ group as older participants tend to have practiced kendo for much longer than average and be of a higher grade level than their peers, however, the causal relationship between kendo experience and better psychometric properties remains unclear. Secondly, as these were all self-report questionnaires we cannot ascertain whether or not they were truthful in their responses nor did we take in to account the circumstances of the participants at the time when answering the questionnaires. We could have added additional questions about daily habitual behaviours and prior incidence of psychiatric illnesses as a way of controlling for extraneous factors but as these self-report questionnaires were conducted online and not in person participants may not be comfortable with providing this information. We also acknowledge that there is a possibility for bias to be present as the individuals who chose to answer the questionnaires may have experienced the benefits of kendo rather than those who do not leading to a possible sampling bias. With regards to individual questionnaires, emotional regulation had a low alpha (0.45) indicating low internal consistency, so this can also be considered a limiting factor. For the Rosenberg self-esteem scale, the trait vs. state hypothesis should also be taken into consideration as it remains uncertain whether or not the participants had these psychometric properties prior to participation in kendo (trait) meaning participation may have had no significant effect on their overall wellbeing. Furthermore, participants current life circumstances outside of kendo can influence their wellbeing too (state) and that may be expressed in their questionnaire answers. The lack of research done on kendo specifically can be considered another limitation as there is not much data to compare with making it difficult to see whether our data is consistent with current findings. In the future, this may be a possible direction, but for now comparisons with martial arts as a whole may be more beneficial instead.

The future perspectives for this study are as such (1) we could do a further analysis of our Rosenberg self-esteem scale data, comparing the average of the positive self-image questions and the average of the negative self-image questions for KPs vs NKPs in both European and Japanese participants. In this way, we can highlight where the significant difference is and further discuss what may be causing this difference. This has been previously done in Sinclair et al., 2010 to compare the different demographic groups living in the United States (Sinclair et al., 2010). (2) Make it a longitudinal study to track participants over a longer period of time and add additional questionnaires that investigate daily life habits and any previous incidence of mental illness in order to further clarify the specificity of kendo’s mental health benefits. (3) Building on from the previous point, recruiting individuals who used to practice kendo habitually and through their questionnaire answers see if they have increased psychometric properties like our participants, if not, is there a time frame in which these benefits last (Table 1).

**Table 1:** Descriptive statistics of both KP and NKP separated by region.

Questionnaires	Region	*N*	Range	Min	Max	Mean	SD
SOC (KP)	EU	164	110	74	184	134.95	22.723
	JP	149	101	74	175	123.52	17.599
SOC (NKP)	EU	37	86	90	176	134.27	24.466
	JP	179	115	64	179	122.48	20.058
ER (KP)	EU	145	25	14	39	26.40	4.634
	JP	137	36	6	42	26.77	5.76
ER (NKP)	EU	35	29	10	39	25.46	5.807
	JP	163	35	7	42	26.04	6.555
K6 (KP)	EU	139	22	0	22	7.67	4.610
	JP	131	23	0	23	6.15	4.651
K6 (NKP)	EU	35	20	0	20	6.97	4.675
	JP	157	24	0	24	5.62	4.620
Self Esteem (KP)	EU	139	25	15	40	30.83	5.280
	JP	130	28	12	40	26.99	5.369
Self Esteem (NKP)	EU	35	23	16	39	30.26	5.972
	JP	157	30	10	40	24.93	5.098
COPE (KP)	EU	136	43	43	86	65.07	9.004
	JP	133	56	29	85	67.92	8.122
COPE (NKP)	EU	35	53	28	81	63.63	12.317
	JP	158	43	45	88	67.03	6.621
CD-RISC (KP)	EU	155	91	2	93	66.26	13.994
	JP	143	93	3	96	54.45	13.539
CD-RISC (NKP)	EU	36	83	10	93	64.83	16.928
	JP	169	89	9	98	52.11	14.037

KP, kendo practitioners; NKP, non-kendo practitioners; K6, Kessler scale 6; COPE, coping orientation to problems experienced inventory; CD-RISC, Connor Davidson Resilience Scale; SOC, sense of coherence; ER, emotional regulation.

In conclusion, this study may be a good addition to research that can help strengthen the connection between habitual martials arts practice and psychometric benefits. Building upon this research a more concrete and robust method can hopefully be established. In the future, it could be an inexpensive alternative to passive therapy for individuals with mental health disorders that may find it difficult to use other present psychotherapy such as standard CBT practices.

CD-RISC, self-esteem, K6 and SOC were all considered significantly higher in KPs than NKPs, supporting the initial hypothesis that kendo players have increased psychometric properties possibly due to core kendo components. Collectively, the recurring significance of self-esteem is interesting as it had already been deemed significant in the Japanese analysis but not in the European analysis possibly due to the suggested cultural differences.

## Data Availability

The raw data supporting the conclusions of this article will be made available by the authors, without undue reservation.
