# Correction: Psychometric properties of martial art kendo players: a multicultural exploratory online questionnaire survey

**DOI:** 10.3389/fpsyg.2025.1722101

**Published:** 2025-10-27

**Authors:** Michael Spantios, Kei Kobayashi, Toshiya Murai, Hironobu Fujiwara

**Affiliations:** Department of Psychiatry, Graduate School of Medicine, Kyoto University, Kyoto, Japan

**Keywords:** mental health, cultural differences, resilience, mindfulness, sport

There was a mistake in Figure 4 as published. Square boxes containing numbers should not appear on the right-hand side. The corrected Figure 4 appears below.

**Figure 4:**
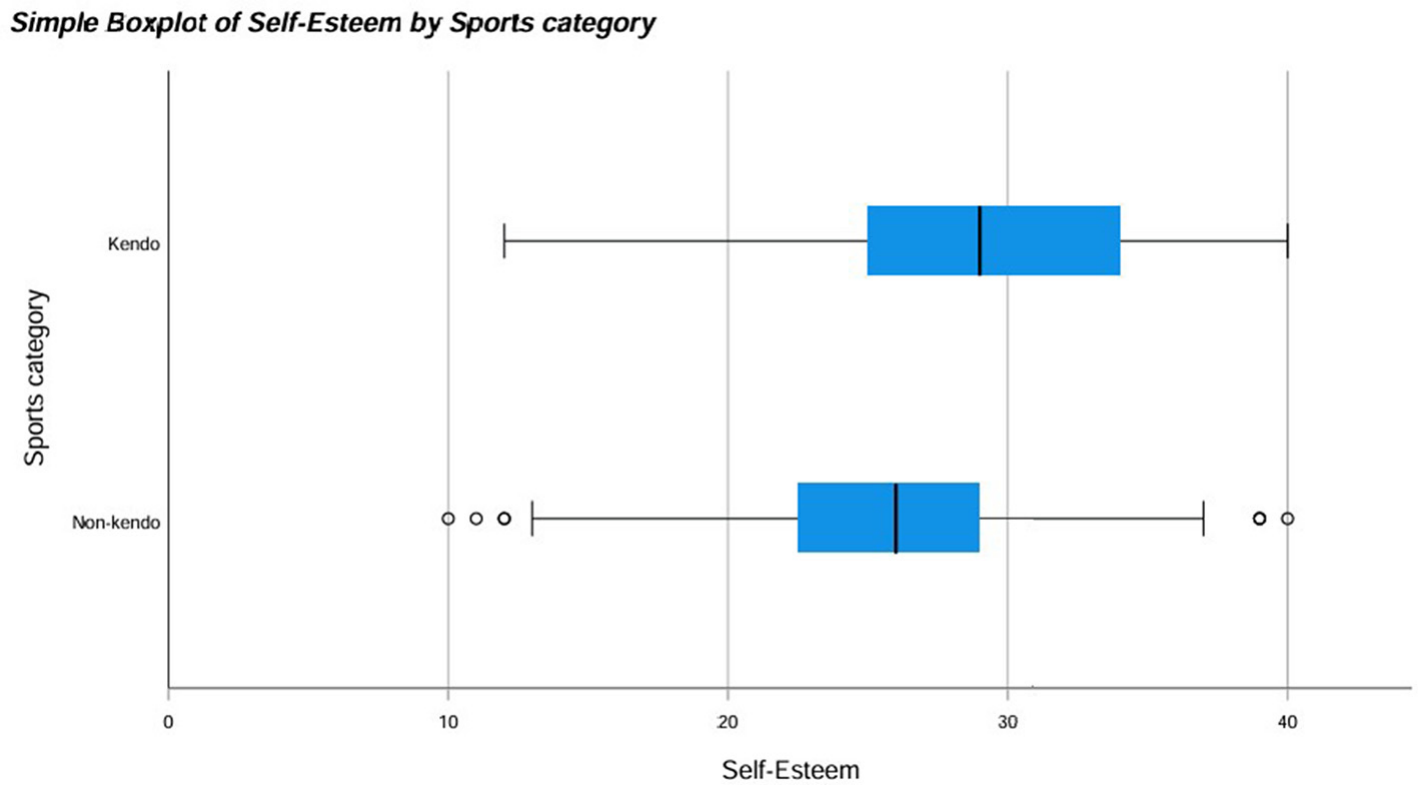
Box plot for self-esteem by sports category including outliers.

The original version of this article has been updated.

