# Supplementary material for: Psychometric properties of martial art kendo players: a multicultural exploratory online questionnaire survey
Source: Front Psychol. 2025 Sep 9;16:1595577. doi: 10.3389/fpsyg.2025.1595577 (PMC12454443; doi:10.3389/fpsyg.2025.1595577)
Supplement: Supplementary file 1 [file Table_1.DOCX]

Supplementary table 1. Participants information

| Western/ Japanese | Num of KP | Num of NKP | Total Participants | Mean Age | Age SD |
| --- | --- | --- | --- | --- | --- |
| Western Men | 147 | 19 | 166 | 40.09 | 14.17 |
| Western Women | 50 | 14 | 64 | 33.92 | 11.30 |
| Total | 197 | 33 | 230 | 38.52 | 13.65 |
| Japanese Men | 102 | 77 | 179 | 34.96 | 13.63 |
| Japanese Women | 55 | 115 | 170 | 26.19 | 10.82 |
| Total | 157 | 192 | 349 | 30.28 | 12.86 |

Note: KP = Kendo Practitioners；Non=Kendo Practitioners
